# Tennis Coaches’ Self-Determined Motivation and Achievement Goals: Links between Coach-Created Motivational Climate, Work Engagement, and Well-Being

**DOI:** 10.3390/bs14080681

**Published:** 2024-08-06

**Authors:** Aristea Kiamouri, Maria Angeli, Charalampos Krommidas, Nikolaos Digelidis, Konstantina Karatrantou

**Affiliations:** Department of Physical Education & Sport Science, University of Thessaly, 42100 Trikala, Greece; akiamouri@uth.gr (A.K.); marangeli@uth.gr (M.A.); hkrom@uth.gr (C.K.); nikdig@uth.gr (N.D.)

**Keywords:** tennis, coach, motivational climate, well-being

## Abstract

Tennis coaches display significant influence, catalyzing changes in athlete performance, motivation, and overall well-being. Research on coaches’ motivations and their impact on coaching style, work, and well-being is limited, especially in individual sports like tennis. Based on self-determination (SDT) and achievement goal (AGT) theories, the aim of the present study was to examine the relationships of coaches’ self-determined motivation (intrinsic, extrinsic, and amotivation), basic psychological needs satisfaction (autonomy, relatedness, and competence), and achievement goals (self-improvement—SI, self-enhancement—SE, self-transcendence—ST) with their coach-created empowering-disempowering motivational climate, work engagement, and well-being (subjective vitality). Participants were 106 tennis coaches from Greece (66 males and 40 females), with an average age of 41.30 ± 12.54 years and coaching experience ranging from six months to 40 years. Data was collected through online questionnaires. Correlation analysis revealed that intrinsic and extrinsic motivation, satisfaction of the three basic psychological needs, and achievement goals were found to be positively related to an empowering climate, work engagement, and subjective vitality. Coaches’ amotivation was positively related to a disempowering climate. Multiple regression analyses showed that autonomy and ST achievement goals significantly predicted tennis coaches’ empowering motivational climate, while none of the independent variables were significant predictors of the disempowering motivational climate. Additionally, intrinsic motivation and ST goal significantly predicted tennis coaches’ work engagement, while autonomy and ST goal significantly predicted their subjective vitality. It is advisable for forthcoming coach education initiatives to consider these findings as an additional justification for tennis coaches to incorporate multiple perspectives into their coaching methodologies.

## 1. Introduction

In the field of organized sports, coaches play a pivotal role in fostering a social environment that forms athletes’ performance, shapes the physical and social development of their athletes, and nurtures their psychological well-being and quality of motivation [1,2,3,4,5]. Coaches’ motivational styles and approaches have been extensively examined through the theories of self-determination (SDT) [6,7,8] and achievement goals (AGT) [9,10].

### 1.1. Self-Determination Theory and Motivation in Sports

SDT attempts to explain why someone exhibits a behavior by providing different types of individuals’ motivation [6]. According to SDT [6,7,8], there are three main types of motivation: (1) Intrinsic (the main reasons for engaging in a behavior are interest, enjoyment [7], and inherent satisfaction); (2) Extrinsic (the primary causes for exhibiting a behavior are compliance, rewards, winning trophies, and punishment); and (3) Amotivation (there is a lack of internal or external motives, an absence of intention to act). Additionally, based on the degree to which motivation is self-regulated by an individual, extrinsic motivation is categorized into four different forms, which are external (e.g., obtain external rewards, avoid punishments), introjected (e.g., avoid guilt or anxiety, enhance self-esteem), identified (e.g., engaging in behaviors that have personal meaning and value), and integrated regulations (e.g., assimilation, alignment with other values and needs of the self) [6,8,11,12]. Amotivation, external regulation, and introjected regulation of extrinsic motivation are the less self-determined types of motivation (controlled motives), while intrinsic motivation, integrated and identified regulation of motivation are the most self-determined forms of motivation (autonomous motives) [7,11].

In addition, SDT [6,7] suggests that individuals’ intrinsic motivation, personal growth, and well-being depends to a large extent on the fulfillment of the three basic psychological needs of autonomy (to act with their own volition, a sense of personal choice, and the ability to make their own decisions), competence (to feel competent, success at challenging tasks, and a sense of efficacy), and relatedness (to feel connected with significant others, to have a sense of belonginess, and feel that they are being cared for by others).

Data from systematic reviews and meta-analyses have shown that the autonomous forms of motivation and the satisfaction of the three basic psychological needs are positively related to children and adolescents’ physical activity levels [13] (autonomous motivation), exercise adherence [14], continued participation in team sports [15], performance at school, work, and physical domains [16] (intrinsic motivation), and better indices of mental and physical health [17]. Furthermore, autonomous forms of motivation and the satisfaction of the three basic psychological needs are negatively related to athletes’ burnout [18] and their intentions to dropout [15]. In contrast, controlled forms of motivation are positively related to poorer mental health [17], athletes’ burnout [18] (amotivation), and intentions to drop out [15]. Controlled forms of motivation (external regulation) are also negatively related to exercise behavior [14].

### 1.2. Achievement Goals and Motivational Climate in Sports

AGT is another theory of motivation that tries to clarify the “beliefs about how success is generally achieved” in different contexts, such as sports or school [10] (p. 290). [19] (p. 312) suggested that people tend to interpret and involve in achievement-related activities based on “the degree to which they tend to judge their competence and define success utilizing task- and/or ego-involved criteria”. According to AGT [9,10], there are two main types of achievement goals defined as task-oriented or mastery goal (emphasis on effort, personal improvement, and collaboration with peers) and ego-oriented or performance goal (emphasis on superiority over others and overcoming others). Recently, Papaioannou and Krommidas [20] introduced a third achievement goal, the self-transcendence goal (ST), which aims to help others improve their competence (e.g., “In Physical Education/sports, my goal is to help my classmates/teammates be more effective than usual”).

A meta-analytical review of Lochbaum et al. [21] in competitive sports found that task-oriented goals are positively related to adaptive success factors, positive emotions, desirable behaviors, autonomous and controlled forms of motivation, perceived competence, self-esteem, task-oriented or mastery motivational climate, and negatively related with amotivation and maladaptive success factors. In contrast, ego-oriented goals are positively related to adaptive and maladaptive success factors, undesirable behaviors, negative emotions, intrinsic motivation, controlled forms of motivation (amotivation, extrinsic, and external regulation), perceived competence, and ego-oriented or performance motivational climate [21]. In competitive tennis, [22] also found that athletes’ task-oriented goals are positively related to their enjoyment, effort, self-talk, goal setting in training and competition perceived performance in competition, and perceived improvement in training. In addition, Papaioannou and Krommidas [20] found that ST goals are strongly related to adolescents’ autonomous motivation, cooperative learning, positive emotions in physical education, and well-being.

Regarding the “atmosphere” created by the coaches during training or competition, Duda [19] suggested that there are two main types of motivational climate created by coaches, termed empowering and disempowering climate. The empowering motivational climate is a combination of an autonomy supportive and a task-oriented climate where coaches emphasize on their athletes’ personal improvement, effort, sense of autonomy and competence, cooperation and teamwork, and are socially supportive [19,23]. In contrast, the disempowering motivational climate is a combination of controlling behaviors and an ego-oriented climate where coaches give emphasize on their athletes’ social comparison (outperforming others) and the final outcome (winning), use controlling behaviors such as shouting, punishment for mistakes, inappropriate comments, and treat their athletes differently depending on their athletic ability [19,23].

### 1.3. The Relationship between Coaching Climate and Athlete Well-Being

A tremendous number of studies have already examined the impact of coach-created motivational climate in various sports. For example, De Clerck et al. [24] indicated that coaches who provide autonomy support and a structuring style tend to boost athletes’ motivation while reducing the autonomy support led to amotivation. Appleton and Duda [23] postulated that coaches who provide an empowering motivational climate tend to enhance athletes’ enjoyment and global self-esteem and reduce their athletic burnout and symptoms of physical illness. Conversely, when they provide a disempowering motivational climate, athletes’ enjoyment and global self-esteem are reduced, while their burnout and physical illness symptoms are boosted. Similarly, Ruiz et al. [25] found that an empowering coach-created motivational climate is positively related to athletes’ happiness, task orientation, and autonomous motivation, while a disempowering coach-created climate is positively associated with increased athletes’ ego orientation, controlled motivation, anxiety, dejection, and anger.

In addition, coaches’ behaviors during training or games can affect their athletes to continue sport or dropout [26,27]. For example, Gillet et al. [26] found that a coach autonomous supportive climate was positively related to athletes’ autonomy motivation, interest and negatively to their intentions to drop out. Similar results were also reported by Sarrazin et al. [27] in handball. Alvarez et al. [28] supported that a task-involving climate positively predicted young soccer players’ satisfaction of the three basic psychological needs, while an ego-involving climate negatively predicted their relatedness satisfaction. Furthermore, basic psychological needs satisfaction positively predicted athletes’ intrinsic motivation, which, in turn, positively influenced their well-being and future intention to continue play soccer [28]. Regarding coach-created motivational climate and athletes’ well-being, similar findings were also reported by Reinboth and Duda [5] in different team sports and Balaguer et al. [29] in youth soccer. In addition, Castillo-Jiménez et al. [30] revealed that an empowering coaching climate was positively related to self-determined motivation, satisfaction of the basic psychological needs, intention to continue sports’ participation, and negatively associated with athletes’ intention to drop out. In contrast, a disempowering climate was positively linked with athletes’ thwarting of the basic psychological needs, intention to drop out, and negatively associated with their self-determined motivation [30].

Recently, a meta-analysis by Mossman et al. [31] found that coaches’ autonomy-supportive climate was positively linked with their athletes’ well-being, autonomous forms of motivation, basic psychological needs for autonomy, competence, and relatedness, performance, engagement, effort, teamwork, and physical activity, as well as other coaches’ behaviors such as task-involving climate, competence and relatedness support, structure, and involvement. Instead, a coaches’ autonomy-supportive climate was negatively related to their athletes’ general ill-being, negative affect, burnout, depression, anxiety, and amotivation, as well as those coaching behaviors that thwarted athletes’ basic psychological needs [31].

### 1.4. Coach Motivation and Well-Being

In addition, several studies have focused on assessing coaches’ motivation using themselves as participants rather than the perceptions of their athletes. In other words, these studies attempted to give an understanding of the “world of coaching” from the coaches’ point of view [32] (p. 346). More specifically, McLean and Mallett [33] showed that connections with sport, coach, and athlete development, external influences, and internal influences are the four main reasons why coaches coach. Rocchi et al. [34] reported that coach motivation and pressure from above (sports administrations) and below (athletes) significantly predicted coaches’ use of autonomy-supportive behaviors. Similarly, Rocchi and Pelletier [35] found that coaching contexts (administrative support, athletes’ motivation, colleague support, time constraints) significantly predicted coaches’ psychological needs satisfaction or frustration, which in turn positively or negatively predicted their autonomous or controlled motivation, respectively. Finally, coaches’ autonomous motivation significantly predicted their psychological need-supportive behaviors, while controlled motivation positively predicted their psychological need thwarting interpersonal behaviors [35]. Guzmán et al. [36] argued that psychological needs satisfaction, perceived competence, and usefulness positively predicted coaches’ adherence and negatively predicted drop-out, while amotivation was an important indicator for dropping out of coaching.

Regarding coaches’ well-being, Stebbings et al. [37] revealed that coaches’ need satisfaction of autonomy and competence positively predicted their psychological well-being, which in turn was a positive determinant of autonomy-supportive behaviors toward their athletes and a negative determinant of their controlling behaviors. Alcaraz et al. [38] revealed that coaches’ intrinsic motivation was positively linked with their subjective vitality, opportunities for professional development, and players’ intrinsic motivation. Furthermore, coaches’ intrinsic motivation was negatively related to amotivation, which in turn was a significant predictor of coaches’ perceived stress [38]. A systematic review of Norris et al. [39] revealed that satisfaction of the basic psychological needs, lack of basic psychological needs thwarting, and self-determined motivation are significant predictors of coaches’ well-being. Similarly, Solstad et al. [40] found that those coaches who adopted more empowering behaviors and lower disempowering behaviors at the beginning of the season also mentioned better well-being at the end of the training season.

### 1.5. Work Environment and Coach Well-Being

Regarding the work environment in sports, Allen and Shaw [32] found that sport organizations were trying to support the basic psychological needs of elite female coaches for autonomy (able to work independently), competence (opportunities and support for formal training, feedback, and mentoring support), and relatedness (clear sense of belonging with other coaches). Stebbings et al. [41] also found that coaches perceptions of higher job security and opportunities for professional development, as well as lower work-life conflict, were positively related to the satisfaction of their basic psychological need, which, in turn, was positively related to coaches’ psychological well-being and their autonomy supportive behaviors toward athletes. Conversely, fewer opportunities for development and higher work–life conflict were positively linked with coaches’ psychological ill-being, psychological needs thwarting, and controlling behaviors [41]. Additionally, Bentzen et al. [42] found that coaches displayed a wide range of burnout symptoms over time due to the frustration of psychological needs and the adoption of a more controlled form of motivation.

### 1.6. The Under-Studied Role of Tennis Coaches

Tennis is an individual sport with more than 87 million players in 41 nations worldwide, which has seen an encouraging development in the total number of tennis courts, clubs and coaches involved in recent years [43]. According to Cakravastia et al. [44], the availability of tennis courts and the presence of certified coaches play an essential role in shaping the number of tennis players within a country.

Although tennis is a global sport and the personal characteristics and behaviors of coaches appear to have a significant impact on players’ motivation and well-being [1,3,4], the different motivational styles adopted by tennis coaches have not yet received significant attention from researchers. For example, Yoo [45] found that a task-involving climate increased students’ tennis skill performance and decreased their levels of anxiety in a physical education setting. Conversely, an ego-involving climate decreased students’ tennis skill performance and retained their state of anxiety [45]. Fry and Newton [46] also supported that a task-involving climate in tennis was positively related to players’ attitudes toward instructor, sportspersonship, and fellow players, while an ego-involving motivational climate in tennis negatively predicted the aforementioned variables. Similar findings in tennis were also reported by Balaguer and colleagues [47] previously. Cervelló et al. [48] found that a task-involving climate in tennis positively related to young athletes’ task orientation, perceptions of a coach-initiated learning motivational climate in competition and their self-assessed performance. Recently, Thorelli [49] found, through semi-structured interviews, that elite Nordic tennis coaches perceived the motivational climate created by the coach in professional tennis as highly ego-involving. Furthermore, studies have consistently shown that when tennis coaches encourage athletes to demonstrate significantly increased levels of enjoyment, sports performance [50] and self-efficacy [51].

Given the significant role of coaches in optimizing athlete performance, psychological development, athletic burnout, and intention to continue sport, research should delve into their needs, experiences, and the impact of their actions on their players [29,35]. In particular, it is important to examine whether tennis coaches’ motivational styles influence their coaching approaches, work engagement, and daily well-being, which in turn may influence their young athletes’ enjoyment and their intention to continue or quit tennis.

Based on the existing literature, the vast majority of the studies focus on how coaching behaviors affect young athletes’ motives, sport participation, and well-being, but not on how coaches’ motives affect their coaching, work, and well-being [32,39,52,53]. Moreover, the number of studies that have used coaches as participants to examine whether their motives can influence their own motivational climate during training or competition or their work environment and well-being seems to be limited in individual sports [33,35,36,37,39,41,42]. Even fewer are those studies that have used tennis coaches as participants to assess their motivational styles [49].

### 1.7. Aim of the Study

The purpose of the present study was to examine whether coaches’ self-determined motivation (intrinsic, extrinsic, amotivation), basic psychological needs satisfaction (autonomy, relatedness, competence), and achievement goals (self-improvement, self-enhancement, self-transcendence) have a significant impact on the coach-created empowering-disempowering motivational climate, work engagement, and well-being (subjective vitality) in daily life.

## 2. Materials and Methods

### 2.1. Participants

Sixty-six male and 40 female Greek tennis coaches (*N* = 106) voluntarily participated in the present study. Their age ranged from 20 to 66 years (*M*_age_: 41.30 ± 12.54 years), while their coaching experience ranged from half a year (six months) to 40 years (*M*_experience_: 12.52 ± 9.67 years). One-hundred and five had a Greek nationality, while one was Cypriot. Eighty-six had a bachelor’s degree in sports science, of which 51 were tennis majors. The remaining 20 coaches obtained their tennis qualifications through seminars and tennis coaching schools.

### 2.2. Instruments

Coach-created motivational climate: A shorter version of the Empowering and Disempowering Motivation Climate Questionnaire (EDMCQ-C), ref. [54] adapted for coaches [55] was administered to tennis coaches to capture their perceptions of the motivational climate they create during training. More specifically, empowering climate were measured with 16 items capturing coaches’ task-involving (nine items, e.g., “During tennis training… I encourage my players to try new skills”), autonomy supportive (four items, e.g., “During tennis training… I give my players choices and options”), and social supportive behaviors (three items, e.g., “During tennis training… I really appreciate players as people, not just as athletes”). Disempowering climate included 14 items capturing the coaches’ ego-involving (six items, e.g., “During tennis training… I give more attention to the best players”) and controlling behaviors (eight items, e.g., “During tennis training… I am less supportive of players when they were not training and/or playing well”). Coaches were asked to indicate their level of agreement on a 7-point Likert scale ranging from 1 (Strongly disagree) to 7 (Strongly agree). The EDMCQ-C has already been used in Greece, during the implementation of the European project “Promoting Adolescents’ Physical Activity” (PAPA); [23], to assess the motivational climate created by the coaches in youth soccer [54,55,56,57].

Self-determined motivation: The Work Tasks Motivation Scale for Teacher questionnaire (WTMST); [58] was used to measure tennis coaches’ self-determined forms of motivation [12]. This measure is composed of fifteen items (the sort of form) that are scored on five subscales (intrinsic, introjected, identified, external, amotivation) [58]. For the purpose of this study, the 15 items scored on three subscales. The first subscale pertained to intrinsic motivation and was assessed through three items (e.g., “I am a tennis coach because … It is pleasant”). The second subscale pertained to extrinsic motivation and included nine items capturing introjected regulation (e.g., “I am a tennis coach because … I would feel guilty not coaching tennis”), identified regulation (e.g., “I am a tennis coach because … I find tennis coaching crucial for the success of my athletes”), and external regulation of motivation (e.g., “I am a tennis coach because … coaching tennis demands it”). Finally, the third subscale assessed amotivation through three items (e.g., “I am a tennis coach because … I used to know why I was coaching tennis, but I don’t see the reason anymore”). Coaches provided their responses on a 7-point Likert scale ranging from 1 to 7, whereby 1 represents “Strongly disagree” and 7 represents “Strongly agree”. The WTMST has already been translated and modified into Greek language for teachers and physical education teachers, e.g., [59,60,61].

Basic psychological needs satisfaction: The Greek version [60,62] of the Need Satisfaction and Frustration Scale (NSFS) [63] utilized to assess tennis coaches’ satisfaction of the basic psychological needs (autonomy, competence, and relatedness). The NSFS includes nine items measuring satisfaction of autonomy (three items, e.g., “When I am on my tennis court… I feel free to decide what to do in tennis training”), competence (three items, e.g., “When I am on my tennis court… I feel highly effective at what I do in tennis training”), and relatedness (three items, e.g., “When I am on my tennis court… I feel very close and connected with other people in tennis club”). Coaches provided their responses on a 7-point Likert scale ranging from 1 to 7, whereby 1 represents “Strongly disagree” and 7 represents “Strongly agree”.

Achievement goals: A shorter version of the Self-Transcendence Achievement Goals Questionnaire (ST-AGQ) [20] was used to examine the orientation of tennis coaches’ goals to improve themselves (self-improvement goals; SI), to outperform others (self-enhancement goals; SE), and to improve others competence (self-transcendence goals; ST). More specifically, the ST-AGQ consisted of nine items capturing tennis coaches’ SI goals (three items; e.g., “In tennis my goal is… do better than the other coaches”), SE goals (three items; e.g., “In tennis my goal is… do better than I typically do”), and ST goals (three items; e.g., “In tennis my goal is… to help the other coaches be more effective than usual”) [20]. The ST-AGQ has already been administered to university students and physical education classes in Greece [20].

Work engagement: The short version of the Utrecht Work Engagement Scale (UWES-9) was used to evaluate tennis coaches’ work engagement levels [64,65]. The nine items of the UWES-9 are divided into three subscales: vigor (three items, e.g., “I feel full of vitality and strength”), dedication (three items, e.g., “Tennis coaching inspires me”), and absorption (three items, e.g., “I am fascinated by tennis coaching”) e.g., [64,65]. Consistent with previous research using different versions of the UWES e.g., [65,66], in the present study all nine items were loaded into a single factor model representing coaches’ work engagement in tennis. Participants were asked to indicate their level of agreement on a 7-point Likert scale ranging from 1 (never) to 7 (always). The full version of UWES, e.g., [64,67], has also been applied to university students, healthcare professionals, and employees in the Greek context, e.g., [68,69,70].

Subjective vitality: Coaches rated their feeling of being alive and energetic using five items (e.g., “I feel alive and vital”) of the Subjective Vitality scale [71]. Coaches were asked to indicate their level of agreement on a 7-point Likert scale ranging from 1 (strongly disagree) to 7 (strongly agree). The Subjective Vitality scale has already been used in Greek language to capture athletes’ well-being e.g., [72,73].

### 2.3. Procedure

The present study was conducted in accordance with the principles of the Declaration of Helsinki and approved by the Ethics Committee of the Department of Physical Education and Sport Science at the University of Thessaly (Ref. Number: 2084; Date of approval: 8 February 2023). The construction and online distribution of the questionnaires to the coaches were carried out using the Google Forms application. Initially, all tennis clubs in Greece were invited by e-mail through the Hellenic Tennis Federation. A researcher then contacted each coach via telephone call. The coaches were informed about the research design, provided their consent, and voluntarily submitted an anonymous online questionnaire with 126 items, including general demographic data. The time necessary to complete the survey was 15 min. The study was conducted between May and October 2023.

### 2.4. Statistical Analysis

All statistical analyses were performed with IBM SPSS v29. The level of significance was set at *p* ≤ 0.05, while the confidence interval (CI) was set at 95%. Initially, descriptive statistics (mean, median, standard deviation), Cronbach’s *α* reliability index, and normal distribution (skewness, kurtosis, Kolmogorov–Smirnov test) of the examined variables were calculated. The vast majority of them did not follow the normal distribution (*p* < 0.05; Table 1). Thus, non-parametric procedures, such as Spearman’s rho correlation or multiple linear regressions with bootstrap estimation, were applied to examine possible links between the continuous variables. Bootstrap estimation, or bootstrapping, is a statistical technique used in research to accurately calculate CI when the data are not normally distributed, e.g., [74,75]. In multiple regression analysis with the bootstrap technique, this is achieved by resampling the observed data at least 1000 times, e.g., [74,75].

## 3. Results

### 3.1. Descriptive Statistics, Reliability Analysis and Normal Distribution

Descriptive statistics (means, standard deviations), Cronbach’s *α* reliability index, and normal distribution (skewness, kurtosis, and Kolmogorov–Smirnov test) of the examined variables are presented thoroughly in Table 1. The vast majority of the data was not normally distributed. All variables showed an acceptable level of reliability (Cronbach’s *α* ranged from 0.71 to 0.95).

### 3.2. Correlation Analysis

Correlation analysis with Spearman rho index revealed that intrinsic and extrinsic motivation, satisfaction of the three basic psychological needs (autonomy, competence, and relatedness), and achievement goals (SI, SE, and ST) were positively related to empowering climate, work engagement, and subjective vitality (except extrinsic motivation; Table 2). Furthermore, all the above variables had negative or no relations with coach-created disempowering climate. In contrast, amotivation had negative or no relations with all the examined variables, except for coach-created disempowering climate where the relationship was positive (Table 2).

### 3.3. Multiple Linear Regression Analyses with Bootstrap Estimation

Separate multiple linear regression analyses with bootstrap estimation were performed to examine whether the independent variables (IVs) of behavioral regulation of motivation (intrinsic, extrinsic, and amotivation), satisfaction of the three basic psychological needs (autonomy, competence, relatedness), and achievement goals (SI, SE, ST) significantly predicted the dependent variables (DVs) of empowering and disempowering coach-created motivational climate. Results revealed that all the IVs explained 52% of the total variance of the empowering climate (*F*_9, 96_ = 11.54, *p* < 0.001; Table 3). More specifically, only autonomy [*B* = 0.19, BC 95% CI (0.10, 0.30)] and ST achievement goal [*B* = 0.13, BC 95% CI (0.02, 0.28)] significantly predicted tennis coaches’ empowering motivational climate, while none of the IVs were significant predictors of disempowering motivational climate, although the regression model was statistically significant (*F*_9, 96_ = 2.90, *p* < 0.01; Table 3).

Similarly, separate multiple linear regression analyses with bootstrap estimation were performed to examine whether the IVs of behavioral regulation of motivation (intrinsic, extrinsic, and amotivation), satisfaction of the three basic psychological needs (autonomy, competence, and relatedness), and achievement goals (SI, SE, and ST) predicted significantly the DV of work engagement. Results revealed that all the IVs explained 39% of the total variance of the work engagement (*F*_9, 96_ = 6.80, *p* < 0.001). More specifically, only intrinsic motivation [*B* = 0.40, BC 95% CI (0.12, 0.69)] and ST achievement goal [*B* = 0.14, BC 95% CI (0.01, 0.30)] significantly predicted tennis coaches’ work engagement (Table 3).

Finally, separate multiple linear regression analyses with bootstrap estimation were performed to examine whether the IVs of behavioral regulation of motivation (intrinsic, extrinsic, and amotivation), satisfaction of the three basic psychological needs (autonomy, competence, relatedness), and achievement goals (SI, SE, ST) predicted significantly the DV of subjective vitality (well-being). Results revealed that all the IVs explained 40% of the total variance of subjective vitality (*F*_9, 96_ = 7.09, *p* < 0.001). More specifically, only autonomy [*B* = 0.38, BC 95% CI (0.05, 0.57)] and ST achievement goal [*B* = 0.25, BC 95% CI (0.11, 0.42)] significantly predicted tennis coaches’ subjective vitality (Table 3).

## 4. Discussion

The objective of this study was to examine the relationship between tennis coaches’ self-determined motivation (intrinsic, extrinsic, and amotivation), satisfaction of basic psychological needs (autonomy, relatedness, and competence), achievement goals (SI, SE, and ST), and the motivational climate created by coaches (empowering or disempowering), in addition to their impact on work engagement and subjective vitality within the context of daily life. As anticipated, tennis coaches guided by intrinsic motives, and who had satisfied basic psychological needs, were found to cultivate an empowering climate, demonstrate engagement in their work, and experience subjective vitality in their daily lives. Norris et al. [39] highlighted that well-being is influenced by the satisfaction of psychological needs and can be transferred to athletes by promoting autonomy-supportive environments. Similar findings by Stebbings et al. [41] underline the impact of the coaching context on coaches’ psychological health and interpersonal behavior toward athletes. It is evident in the literature that a coach-created motivational climate influences athletes’ capacity development, simultaneously potentiating and exposing their vulnerabilities [6].

In light of the current cross-sectional study’s findings, the behavioral regulation of motivation (intrinsic, extrinsic, and amotivation), the satisfaction of the three basic psychological needs (autonomy, competence, and relatedness), and achievement goals (SI, SE, and ST) emerged as significant predictors of the empowering and disempowering coach-created motivational climate for tennis coaches. Findings align with relevant studies [30,36,76], underscoring the importance of fostering an empowering motivational climate and preventing the fostering of a disempowering climate. More specifically, Castillo-Jiménez et al. [30], suggested that need satisfaction boosts the intention to continue sports, while need thwarting directly increases dropout intentions.

The satisfaction of their basic psychological needs leads to a positive influence on athletes. More specifically, Stebbings et al. [77] suggested that enhancing coaches’ psychological well-being can foster a more adaptive interpersonal environment for athletes. The coach’s well-being is crucial, as it directly impacts their ability to lead, inspire, and guide. The bonds between types of motivation, satisfaction of the three psychological needs, and the types of achievement goals are significant for a coach to foster either an empowering or disempowering climate by influencing the overall environment.

Despite correlations observed among all the variables, results showed that only autonomy and ST achievement goals significantly predicted tennis coaches’ empowering motivational climate. Since the ST achievement goal, rooted in empathy and concern for others, has been linked to autonomous motivation [20], it is unsurprising that both constructs predict an empowering climate. However, amotivation indicated a positive relationship with a coach-created disempowering climate. A disempowering climate often stems from coaches’ amotivation, leading to a lack of autonomy, competence, and relatedness for athletes [18,23]. Amotivation is identified as an important indicator for coaches dropping out of their roles [36]. Therefore, it is reasonable to posit a positive relationship between a disempowering climate and amotivation, which denotes a lack of intention to act or a motivation deficit [7]. Autonomy motivation and ST achievement goals were both highly predictive of the subjective vitality of tennis coaches. These findings are promising to support prior evidence by Papaioannou and Krommidas [20], who postulated that ST achievement goals are autonomous-oriented and positively linked with subjective vitality.

Intrinsic motivation and ST achievement goals significantly predicted the work engagement of tennis coaches. The concept of self-transcendence, which involves transcending personal concerns and connecting with a larger purpose, is a goal closely related to individuals’ autonomous motivation, cooperative learning, positive emotions in PE, and overall well-being [20]. In addition, volitional action to benefit others [78] has been found to be positively related to autonomy-supportive teaching [79]. Regarding the coaching approach, research by Lundkvist et al. [80] indicates that soccer coaches who maintain a good work-life balance and have access to well-being resources report higher levels of job satisfaction and engagement.

Hence, these findings are consistent with Vallerand’s [81] assertion that intrinsic motivation influences engagement and commitment within the coaching context, since intrinsic motivation and achievement goals are significant predictors of work engagement. It is interesting to note that intrinsic motivation fosters enthusiasm, commitment, and perseverance [82]. However, according to Ntoumanis and Biddle’s [83] review, the influence of achievement goals on engagement is discussed.

Despite its importance, coaching remains a relatively underexplored and poorly understood field [84]. Overall, the findings of this study are valuable for designing coach education programs aimed at producing high-quality players capable of competing internationally [44]. Our findings are equally valuable for developing and implementing theory-based interventions [85], designed to modify the tennis coaching climate (see Empowering Coaching™) [19], thereby altering coaches’ motivation and influencing their teaching styles.

## 5. Limitations

The generalizability of these findings is limited by the sample’s heterogeneity in terms of gender, age, and experience. Future research with larger, more representative samples could explore potential gender or experience effects. Another important limitation of the present study is the lack of data analysis based on coaches’ degrees of certification or specialized training. Even with extensive experience, a coach may only hold a first-level education certificate. Besides practical experience, academic programs and the acquisition of new knowledge and information through specialized seminars may have a greater impact on coaches’ professional growth and their athletes’ performance. Furthermore, exploring the influence of tennis club characteristics (coaches’ perceptions of their club environment) on coach motives, work engagement, and well-being could provide a more comprehensive understanding.

## 6. Conclusions

This study offers a comprehensive examination of the influence of intrinsic motivation, autonomy satisfaction, self-transcendent goals, and experience on empowering climate, work engagement, and subjective vitality among tennis coaches. The hypothesis that motivation-weighted behavior predicts an empowering climate, work commitment, and well-being was confirmed. Based on the above results, it appears that there are similarities between the motivational styles displayed by tennis coaches and those of other individual or team sports. Fostering autonomy and prosocial motives in tennis coaches is essential for creating positive work environments. Motivation itself, whether autonomous or controlled, leads to diverse cognitive, emotional, and behavioral outcomes. Interventions promoting these qualities in coaches could include emphasizing self-improvement, commitment, teamwork, and providing regular feedback. Identifying coaches’ motivational profiles can inform interventions promoting autonomy, ST goals, and intrinsic motivation, ultimately enhancing coach effectiveness in dynamic environments. This approach can foster an empowering climate, potentially leading to increased work engagement with tennis and subjective vitality.

## Figures and Tables

**Table 1:** Descriptive statistics (means, standard deviations), Cronbach’s α reliability index, and normal distribution (skewness, kurtosis, Kolmogorov–Smirnov test) of the examined variables.

Variables	*M*	*SD*	*α*	Skewness	Kurtosis	K–S *p*-Value
Empowering climate	6.16	0.64	0.91	−0.83	0.91	0.021
Disempowering climate	3.01	1.07	0.87	0.93	1.87	0.146
Intrinsic motivation	6.54	0.55	0.80	−1.43	2.52	0.000
Extrinsic motivation	4.31	0.92	0.76	−0.11	−0.22	0.200
Amotivation	1.77	0.99	0.76	1.66	3.27	0.000
Autonomy	5.98	1.01	0.84	−0.92	0.14	0.000
Competence	5.60	0.98	0.89	−0.61	−0.06	0.000
Relatedness	5.47	1.11	0.76	−0.50	−0.64	0.000
SI goal	6.48	0.62	0.75	−1.12	0.60	0.000
SE goal	4.66	1.56	0.71	−0.64	−0.19	0.000
ST goal	5.64	1.30	0.94	−1.05	0.52	0.000
Work engagement	5.76	0.98	0.95	−1.09	2.29	0.008
Subjective vitality	5.45	1.02	0.92	−0.38	−0.03	0.124

Note. SI = self-improvement achievement goal; SE = self-enhancement achievement goal; ST = self-transcendence achievement goal; *M* = mean; *SD* = standard deviation; *α* = Cronbach’s *α* reliability index; K–S = Kolmogorov–Smirnov test.

**Table 2:** Spearman’s correlation analysis between the examined variables.

Variables	1	2	3	4	5	6	7	8	9	10	11	12	13
1. Empowering climate	-												
2. Disempowering climate	−0.26 **	-											
3. Intrinsic motivation	0.48 ***	−0.08	-										
4. Extrinsic motivation	0.25 **	0.16	0.36 ***	-									
5. Amotivation	−0.34 ***	0.21 *	−0.37 ***	0.19	-								
6. Autonomy	0.57 ***	−0.07	0.61 ***	0.28 **	−0.22 *	-							
7. Competence	0.41 ***	−0.12	0.47 ***	0.30 **	−0.19	0.56 ***	-						
8. Relatedness	0.40 ***	−0.06	0.42 ***	0.27 **	−0.16	0.50 ***	0.61 ***	-					
9. SI goal	0.56 ***	−0.09	0.43 ***	0.12	−0.43 ***	0.47 ***	0.38 ***	0.39 ***	-				
10. SE goal	0.15	0.19	0.17	0.25 **	0.09	0.29 **	0.32 ***	0.17	0.14	-			
11. ST goal	0.63 ***	0.00	0.43 ***	0.22 *	−0.26 **	0.46 ***	0.45 ***	0.44 ***	0.55 ***	0.37 ***	-		
12. Work engagement	0.62 ***	−0.10	0.55 ***	0.27 **	−0.30 **	0.50 ***	0.50 ***	0.41 ***	0.42 ***	0.21 *	0.53 ***	-	
13. Subjective vitality	0.48 ***	−0.11	0.40 ***	0.18	−0.17	0.46 ***	0.50 ***	0.28 **	0.22 *	0.19	0.44 ***	0.76 ***	-

Note. SI = self-improvement achievement goal; SE = self-enhancement achievement goal; ST = self-transcendence achievement goal; * *p* < 0.05; ** *p* < 0.01; *** *p* < 0.001.

**Table 3:** Results of bootstrapped multiple linear regression analyses with coach-created motivational climate (empowering and disempowering), work engagement, and subjective vitality as DVs.

DVs	IVs					Bootstrap ^a^				
		*R*	*R* ^2^	*F*	*B*	Bias	SE	*p*	BC 95% CI	
									LL	UL
Empowering climate	(Constant)	0.72	0.52	11.54 ***	2.42	0.10	0.89	0.005	0.68	4.37
	Intrinsic motivation				0.05	−0.02	0.12	0.654	−0.18	0.22
	Extrinsic motivation				0.07	0.00	0.06	0.254	−0.04	0.18
	Amotivation				−0.09	−0.00	0.06	0.132	−0.20	0.01
	Autonomy				0.19	0.00	0.05	0.001	0.10	0.30
	Competence				0.03	0.00	0.07	0.665	−0.11	0.18
	Relatedness				−0.02	−6.37	0.05	0.702	−0.13	0.09
	SI goal				0.23	−0.00	0.13	0.095	−0.02	0.48
	SE goal				−0.02	−0.00	0.03	0.426	−0.09	0.03
	ST goal				0.13	0.01	0.06	0.040	0.02	0.28
Disempowering climate	(Constant)	0.46	0.21	2.90 **	1.11	0.04	1.61	0.507	−2.23	4.29
	Intrinsic motivation				0.14	6.52	0.23	0.501	−0.29	0.59
	Extrinsic motivation				0.15	−0.01	0.14	0.259	−0.10	0.38
	Amotivation				0.31	−0.00	0.15	0.045	−0.00	0.61
	Autonomy				−0.06	−0.02	0.16	0.688	−0.40	0.19
	Competence				−0.31	0.03	0.20	0.138	−0.69	0.16
	Relatedness				−0.00	0.01	0.13	0.973	−0.23	0.25
	SI goal				0.11	0.00	0.20	0.576	−0.24	0.51
	SE goal				0.14	−0.01	0.08	0.069	0.00	0.27
	ST goal				0.09	−0.01	0.09	0.317	−0.08	0.24
Work engagement	(Constant)	0.62	0.39	6.80 ***	−0.26	0.07	1.26	0.845	−2.65	2.48
	Intrinsic motivation				0.40	−0.01	0.17	0.025	0.12	0.69
	Extrinsic motivation				0.01	−0.01	0.13	0.944	−0.28	0.20
	Amotivation				−0.07	0.01	0.10	0.452	−0.27	0.16
	Autonomy				0.17	0.00	0.12	0.128	−0.01	0.43
	Competence				0.14	−0.00	0.12	0.253	−0.08	0.36
	Relatedness				−0.03	0.00	0.08	0.708	−0.18	0.14
	SI goal				0.15	−0.01	0.17	0.350	−0.16	0.48
	SE goal				0.03	0.00	0.06	0.646	−0.07	0.15
	ST goal				0.14	0.00	0.07	0.053	0.01	0.30
Subjective vitality	(Constant)	0.63	0.40	7.09 ***	2.26	0.01	1.25	0.072	−0.13	4.73
	Intrinsic motivation				0.13	−0.00	0.18	0.447	−0.21	0.49
	Extrinsic motivation				0.03	−0.01	0.12	0.836	−0.22	0.22
	Amotivation				−0.08	0.02	0.10	0.445	−0.27	0.19
	Autonomy				0.38	−0.03	0.15	0.009	0.05	0.57
	Competence				0.28	0.01	0.15	0.062	0.01	0.60
	Relatedness				−0.16	0.02	0.10	0.123	−0.37	0.09
	SI goal				−0.29	0.01	0.16	0.069	−0.59	0.05
	SE goal				−0.03	0.00	0.05	0.560	−0.14	0.08
	ST goal				0.25	0.00	0.08	0.005	0.11	0.42

Note. ^a^ Results with bootstrap estimation are based on 2000 bootstrap samples; IVs = independent variables; DVs = dependent variables; SE = standard error; BC = bias corrected; CI = confidence interval; LL = lower limit; UL = upper limit; ** *p* < 0.01; *** *p* < 0.001; SI = self-improvement achievement goal; SE = self-enhancement achievement goal; ST = self-transcendence achievement goal.

## Data Availability

The data presented in this study are available on request from the corresponding author. The data are not publicly available due to privacy/ethical restrictions.
